# Stress induced telomere shortening: longer life with less mutations?

**DOI:** 10.1186/1752-0509-8-27

**Published:** 2014-03-01

**Authors:** Ala Trusina

**Affiliations:** 1 Niels Bohr Institute, University of Copenhagen, Blegdamsvej 17, DK 2100, Copenhagen, Denmark

**Keywords:** Telomere shortening, Reactive Oxygen Species (ROS), Cell-to-cell heterogeneity, Genotoxic stress, Mathematical model

## Abstract

**Background:**

Mutations accumulate as a result of DNA damage and imperfect DNA repair machinery. In higher eukaryotes the accumulation and spread of mutations is limited in two primary ways: through *p53-mediated* programmed cell death and *cellular senescence* mediated by telomeres. Telomeres shorten at every cell division and cell stops dividing once the shortest telomere reaches a critical length. It has been shown that the rate of telomere attrition is accelerated when cells are exposed to DNA damaging agents. However the implications of this mechanism are not fully understood.

**Results:**

With the help of *in silico* model we investigate the effect of genotoxic stress on telomere attrition and apoptosis in a population of non-identical replicating cells. When comparing the populations of cells with constant vs. stress-induced rate of telomere shortening we find that stress induced telomere shortening (SITS) increases longevity while reducing mutation rate. Interestingly, however, the effect takes place only when genotoxic stresses (e.g. reactive oxygen species due to metabolic activity) are distributed non-equally among cells.

**Conclusions:**

Our results for the first time show how non-equal distribution of metabolic load (and associated genotoxic stresses) combined with stress induced telomere shortening can delay aging and minimize mutations.

## Background

Mutations accumulate as a result of DNA damage – an unavoidable byproduct of life: damage to DNA is caused by metabolic activity [1,2], DNA replication [3], exposure to UV light, etc. Most of the damage is rapidly and successfully repaired by complex DNA repair pathways [4]. However the fidelity of the repair proteins and pathways is not perfect and erroneously repaired or unrepaired damage can result in mutations [5].

There exist two main ways to limit mutation accumulation in the population of cells: a) eliminate damaged cells or b) set a limit on the number of replications a cell can undergo. In the former case, a severely damaged cell can be removed form the pool of replicating cells in many ways: it can undergo cell-cycle arrest, autophagy, necrosis or activate pre-programmed suicidal program – apoptosis. This decision making is mediated at the level of a *single cell* and occurs within *hours* from the initial insult. Nearly all these processes involve p53 – a master regulator protein [6]. In the following sections, for simplicity, the combined effect of the p53 and other proteins regulating removal of severely damaged cells will be referred to as p53.

In an alternative scenario b) the spread of mutations in a *lineage* of proliferated cells is limited by telomeres and happens on the time-scale of *weeks*. The number of replications a given proliferated cell can undergo is given by the length of its telomeres – a stretch of (TTAGGG) DNA repeats at the chromosomal ends. Proliferated cells start with a wide distribution of telomere lengths (with the average about 15000 bp in e.g. human fibroblasts [7])) which progressively shorten at every cell division. Once the shortest telomere reaches the critical length (e.g. of about [8] 2000 bp in human fibrobasts), cells undergo replicative senescence – they stop dividing and in some cases undergo apoptosis.

It has been shown that the rate of telomere shortening is accelerated when cells are exposed to genotoxic stresses (e.g. reactive oxygen species (ROS)) [9]. These results shift the telomere paradigm from a simple clock counting cell divisions to a more sophisticated device recording the *history of stress exposure* within a cell lineage. While these results have opened a new perspective on replicative senescence, it is still unclear how and under what conditions can cells benefit from such a mechanism? As p53 already removes damaged cells that have a high chance to accumulate mutations, what does one gain by Stress-Induced Telomere Shortening (SITS) compared to classical view of Telomere Shortening (TS) at a constant rate?

To answer these questions we introduce a semi-quantitative model of replicating cells exposed to non-uniform genotoxic stresses.

## Methods

### Model

The model consists of population of replicating and dying cells. The population is limited to 400 replicating cells: when the population size drops below 400 a random cell, that has divided more than 24 hours (set to be cell doubling time) prior to present event, is picked and is allowed to divide. In the absence of genotoxic stress, cells are dying stochastically (due to DNA damage unrelated reasons) with a constant rate *β*_0_=0.02 such that on average each cell doubles every 24 hours. To easier relate our model to the experimental data we choose to report our results in units of “cell doublings” with one cell doubling being equivalent to 24 hours in our model. The population starts with 400 replicating cells having initial telomere length of 15000 bp. Cells are removed from the pool of replicating cells if either telomere length is below critical length (2000 bp) or the p53 mediated mechanisms are activated. In the following sections we present the biological basis for our model, followed by the detailed description of the model implementation.

#### Biological basis of the model

**Figure 1:**
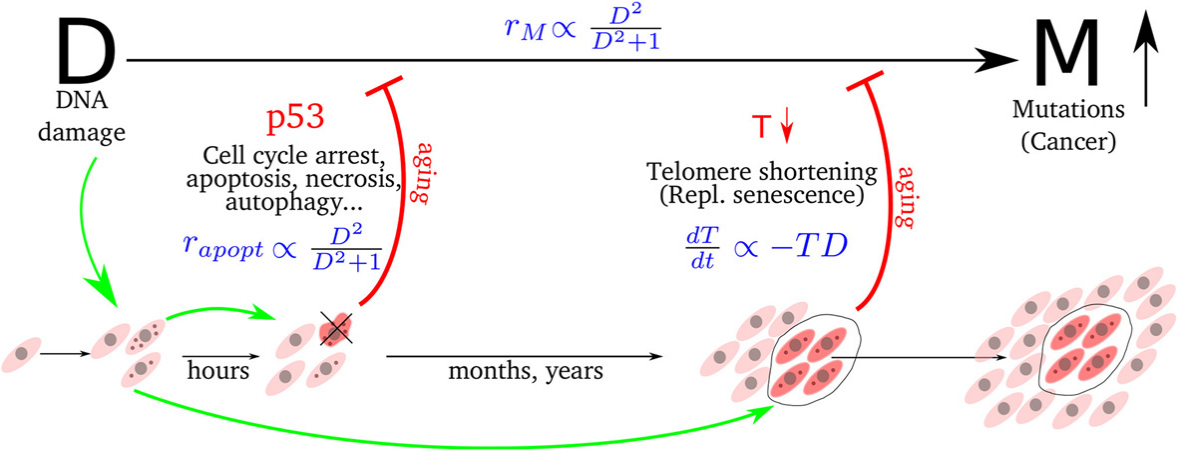
**Schematic diagram picturing the inter-relations between DNA damage, cellular aging and cancer.** As a result of DNA damage (D), induced by genotoxic stresses, mutations accumulate in individual cells (M). Cells exposed to larger amounts of stress have higher probability to accumulate more mutations. The replication of these potential mutators (i.e. tumor originating cells), is limited by the two distinct aging mechanisms: a) replicative senescence mediated by telomere attrition and b) p53 mediated cell cycle arrest and apoptosis. While the effect of p53 is rapid (can happen within several hours after the insult), the effect of telomeres is slow.

The main components of the model are: i)DNA damage, *D*, ii) accumulation of mutations, *M*, iii) cell cycle arrest, apoptosis and other p53 mediated responses, *p*53 and iv) telomere shortening, *T* (see Figure 1).

i) The level of *DNA damage*, *D*: is assigned from a normal distribution with a given average *μ*_*D*_ and a standard deviation *σ*_*D*_. The variation in *D* might arise from multiple sources: fluctuations in concentrations of DNA repair enzymes, variations in metabolic load (and subsequent Reactive Oxygen Species (ROS) production) in single cells, etc. The particular choice of the shape of the distribution (e.g. it has been shown that cell-to-cell variation in gene expression sometimes follows lognormal distribution [10,11]) does not qualitative change the main outcomes of the model.

As the time-scale relevant for our simulation is of the order of cell division (characteristic time-scale for both telomere decay and mutation rate) we assign a new value of *D* every time cells divide. In fact any fluctuations much faster than cell doubling time will be averaged out and result in a “homogeneous population” where each cell experiences the same damage seen at the time-scale of cell doubling. The other limit, when fluctuations in D are much slower than doubling time, will again result in somewhat “homogeneous population” with several different groups of cells. Thus the most interesting regime is when *D* changes on time-scale of cell doubling. Note that while we are assigning the damage from Gaussian distribution, the resulting distribution of damage in the simulated population of replicating cells can be different from Gaussian (e.g. damage can not be negative). In the following we will denote the damage averaged over cells and time as 〈*D*〉.

ii) *Mutations*, *M* spread and accumulate in the population as mutated cells replicate. Cells with many mutations have higher chance to originate tumor cells [12] and can be thought of as tumor progenitors.

The genotoxic stress, e.g. oxidative stress, replication of DNA fragile sites, UV or gamma radiation, etc. results in DNA damage (D, Figure 1). Damaged DNA recruits DNA repair machinery by e.g. activating ATM signaling cascade. In most of cases DNA repair enzymes remove the damage [13-15], however the repair is not perfect and often mutations occur as a result of damage and repair cycles. Thus higher genotoxic stress leads to more mutations [5]. We model this dependence by setting the rate of probability for a mutation to occur,

(1) $$r_{M}=\alpha \frac{D^{2}}{D^{2}+1}.$$

The typical mutation rate is estimated to be 10^-11^ (somatic stem cells) - 10^-9^ (typical for proliferated cells) per basepair per cell division, which amounts to 0.01-1 per human cell per cell division [12]. As we are simulating observations made in proliferated cells, we set *α*=1 per cell division. We choose mutation rate of *r*_*M*_=0.2 per cell per cell division to represent “typical” mutation rate under “physiological” damage (thus with *α*=1 the range of “physiological” damage is 〈*D*〉∼0.5). The main results will be qualitatively the same if the mutation rate is increased or decreased 5 fold.

iii) *p*53-mediated responses limit the mutation spread by rapidly (compared to the telomere attrition) eliminating stressed cells. Cell survival under DNA damage have sigmoid dose-response curve [16], we have modeled this observations by setting probability for p53 mediated cell death to be a sigmoid curve

(2) $$r_{\text{apopt}}=\beta \frac{D^{2}}{D^{2}+1}$$

Observe that the functional form in mutational and apoptotic probabilities are set to be the same as this allows most efficient elimination of mutated cells. Parameter *β*=0.1 is chosen such that just a small fraction of cells (0.1%) undergoes apoptosis at low levels of *D*∼0.2.

The results of the model do not depend on the choice of the functional forms for probabilities to mutate or undergo apoptosis. (See Additional file 1: Figure S1.)

iv) Telomeres consist of (TTAGGG) repeats which form a protective cap at the end of eukaryotic chromosomes. During cells division, the 3’ end of linear DNA can not be fully replicated and thus telomeres become shorter. Cells with short telomeres (2000 bp) lose the ability to replicate and at this stage they either exist in a non-dividing state or undergo programmed cell death. Interestingly, the rate of loss of telomeric DNA is not constant but appears to depend on the length of telomere [17,18] and the level of oxidative stress [9]. In our model we capture these observation by setting the telomere decay to be proportional to DNA damage, *D*[8] and the length of telomere, *T*[17,18].

(3) $$\frac{\text{dT}}{\text{dt}}=-\mathrm{\gamma TD}$$

Thus we do not explicitly model the mechanism of how telomeric damage leads to telomere decay. This has been carefully addressed in the model by Proctor et al. [8] and is beyond the scope of our work. Instead we phenomenologically describe the observed correlation between the rate of telomere decay and DNA damage in the cell and assume that the cellular damage is independent of telomere length. While we model one telomere per cell, in reality there 92 telomeres per cell. Telomere lengths follow a skewed, lognormal-like, distribution and it is believed that the the replicative senescence is dictated by the shortest telomere [19]. Furthermore, we model telomere decay as a continuous process while in living cells the decrease in telomere length is related to the replication and happens at cell division. Replacing continuous update of telomere lengths with a discrete update leads to same qualitative results.

Initial telomere length in human fibroblasts was estimated to be 15000 bp and the rate of decay is about 100 bp per division [7]. The exact values of the initial and critical telomere lengths do not affect the qualitative results of the model. Parameter *γ*=1.5×10^-3^ is given by the requirement of decay of 50-100 bp per cell division when DNA damage, D is low (*D*=0.2). This description of telomere dynamics is inspired by the model by Proctor et al. [8]. As parameters *α*,*β* and *γ* are constrained by experimental data, the only free parameters of interest are average DNA damage, 〈*D*〉 and how the damage differs from cell-to-cell, *σ*_*D*_.

#### Model execution

The code executing the model is programed in C++ and completes within minutes on a standard PC. For each cell we keep track of the following attributes: 

*D* DNA damage, assigned at cell division from gaussian distribution with *μ*_*D*_ and *σ*_*D*_

*t*_*birth*_ birth time, set for daughter cells after every division

*τ*_*surv*_ survival time, assigned at cell division. To arrive to a damage-induced rate of cell death given by Eq. 2 and account for stochastic damage-unrelated death with rate *β*_0_, *τ*_*surv*_ is drawn from exponential distribution $e^{-\beta_{0}-\beta D^{2}/(D^{2}+1)(t-t_{\text{birth}})}$.

*T* Telomere length, updated every time step.

*n*_*mut*_ mutation counter, updated every time step.

The time evolution of the model is as follows: 

**0)** At time *t*=0, for each cell among 400 cells we initialize *T*=15000, *n*_*mut*_=0, *t*_*birth*_=0 and assign *D* and *τ*_*surv*_ as described above.

**1)** At every time step advance in time with *dt*=0.1*h* and in each cell 

- Update *T*, according to Eq.3.

- Increase *n*_*mut*_ by one with probability given by Eq. 1

- If *T*≤2000 or *t*_*current*_-*t*_*birth*_≥*τ*_*surv*_, i.e. if cell turns senescent or undergoes apoptosis 

* remove cell from the population of dividing cells.

* Divide a cell chosen randomly among those with *t*_*current*_-*t*_*birth*_≥24.

* Daughter cells inherit *T*,*n*_*mut*_ and get assigned new *D* and *τ*_*surv*_ as described above. For each of the daughter cells set *t*_*birth*_=*t*_*current*_.

**2)** Repeat advancing in time as described in **0)** until there are no replicating cells left in the population.

## Results and discussion

The main objective of our model is to investigate the effect SITS has on mutation accumulation in a population of replicating cells. We hypothesized that the mechanism of SITS, which specifically accelerates senescence in more damaged progenies, will probably result in a slower mutation rate than in case of a constant, i.e. stress independent telomere shortening (TS). (In SITS the more damaged cells and thus ones with higher number of mutations, will be the first to undergo senescence. Thus SITS removes mutations from the population at a higher rate than TS.)

### SITS increases longevity while minimizing mutation rate

**Figure 2:**
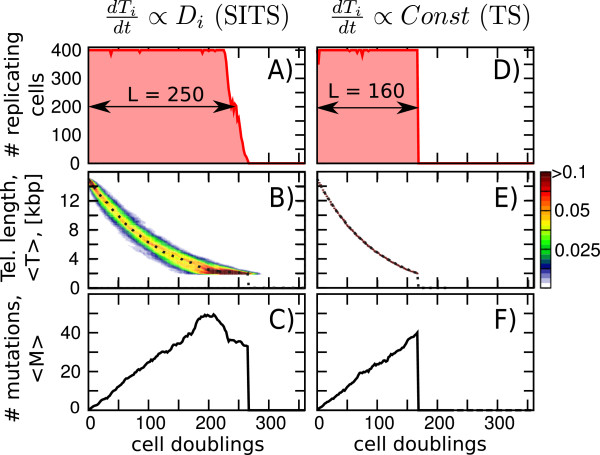
**Stress-Induced Telomere Shortening (SITS) (left, A-C) allows for delayed aging when compared to the constant rate of Telomere Shortening (TS) (right, D-F). A)** and **D)** show how the number of cells capable of replication change in time. In both cases, as cells divide, the average telomere length, <*T*>, decreases (**B)** and **E)**) and the number of accumulated mutations, <*M*> increases (**C** and **F)**). In **B)** and **E)** The colorcoded are the distribution of telomere lengths in the population, the average is shown by dotted line. Observe that in **C)** (but not in **F**) the rate of increase in *M*, *dM*/*dt* is slowing down at later time points. The results are shown for DNA damage <*D*> =0.25 and the cell-to-cell variation in DNA damage, $\frac{\sigma_{D}}{<D>}=1.5$. For TS case the constant rate of telomere shortening *Const*=0.25.

In Figure 2 we show the dynamics of the three main characteristics: Number of dividing cells in population, *N*, telomere length, *T* and number of mutations, *M* averaged over all cells. When comparing the dynamics of SITS (Figure 2A-C) and TS models (Figure 2D-F) we find two remarkable results: a) SITS indeed decreases mutation rate but the effect is taking place at late time points (Figure 2 C and F) and b) SITS significantly increases the longevity of population, *L*, defined as number of replications after which the population drops below 200 (compare Figure 2A and D).

Both the increase in longevity and decrease in mutation rate are late events that take place when population of cells approaches a critical length in their telomeres (known as Hayflick limit [20]). The SITS produces wide distribution of telomere lengths. When average telomere length approaches the Hayflick limit cells with telomeres longer than the limit divide to compensate for dying cells with critically short telomeres, thus the average telomere length can be maintained above the limit for longer time. In Figure 2B this phenomena happens at around t = 150 cell divisions.

At early time points – when the mutation rate is determined only by the amount of DNA damage, <*D*>, in the population of cells and the apoptosis rate – there is no difference between the two scenarios. However at later time, when population starts approaching Hayflick limit (e.g. at t = 150), in SITS (but not in TS) a considerable amount of cells with short telomeres (i.e. stressed cells that accumulated many mutations) has been replaced by cells with longer telomeres and few mutations. Observe that the constant mutation rate over the whole life span would result in linear increase in number of mutations *M* with increasing longevity *L*. The SITS allows to break this linear dependence at late time points.

It is important to note that we have chosen the rate of telomere shortening to depend only linearly on the DNA damage, i.e. $\frac{dT_{i}}{\text{dt}}∼D_{i}$, the effect on mutation rate would be even stronger if we use square or higher powers as this will make telomeres differentiate even more between damaged and non-damaged cells. Interestingly Zglinicki et al. [9] reported nearly square dependence of the rate of telomere shortening on the amount of ROS in different cell lines.

As the metabolic load varies widely from one cell type to another, as well as between individual cells in the population we wanted to investigate how our results depend on the mean DNA damage <*D*> and the cell-to-cell variability.

**Figure 3:**
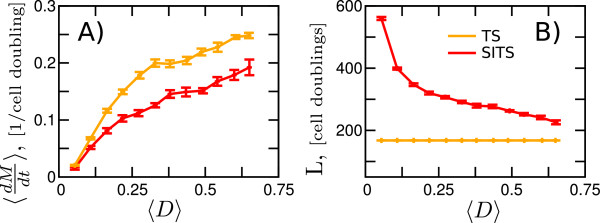
**The SITS mediated gain (combined decrease in mutation rate and increase in longevity) is maximal at intermediate levels of DNA damage.** The time average of the mutation rate, $\langle \frac{\text{dM}}{\text{dt}}\rangle$**(A)** and longevity, *L***(B)** are shown as function of DNA damage, 〈*D*〉. Each point represents an average of 100 simulation runs. To scan across increasing average DNA damage, 〈*D*〉, we altered the mean of the gaussian distribution, *μ*_*D*_. The red(orange) lines and corresponding errorbars represent SITS (TS). Cell-to-cell variation in DNA damage, $\frac{\sigma_{D}}{\langle D\rangle }=1.5$.

In Figure 3A we monitor the mutation rate, $<\frac{\text{dM}}{\text{dt}}>$ averaged over time and cells. Due to p53 mediated apoptosis, cells with DNA damage, *D*≫1 will be removed from population and thus the average of the assigned DNA damage, *μ*_*D*_ will differ from the actual average DNA damage, 〈*D*〉, averaged over replicating cells. We choose to use the latter as it allows us to focus on telomere effect alone.

While both SITS and TS mutation rates increase with increasing genotoxic load, 〈*D*〉, the SITS slows down the increase for the low and intermediate levels of 〈*D*〉∼0.025-0.375. The beneficial effect of SITS is maintained for all analyzed 〈*D*〉, but it saturates at higher levels of DNA damage, as p53 eliminates more and more cells.Remarkably, while in SITS case, longevity is decreasing with increasing DNA damage, it always remains above the TS values (see Figure 3B). The combined gain in increased longevity and decreased mutation rate is maximal at intermediate values of DNA damage, in the range between the “physiological” and apoptotic loads of DNA damage.

### Cell-to-cell variability is required for SITS to take an effect

As we have seen in Figure 2B, both the increase in longevity as well as damped mutation rate rely on a wide distribution of telomere lengths. In our model the distribution of telomere lengths during SITS originates from the cell-to-cell variation in DNA damage. (If all cells were subject to the same level of DNA damage, all cells would have the same telomere length). Interestingly the wide distribution of telomere lengths can result from Telomeric Sister Chromatid Exchange (T-SCE) [21].

Antal et al., [22] have elegantly analyzed this phenomena treating it analytically as a diffusion-convection problem. They show that increasing the rate of T-SCE (while maintaining the same telomere attrition rate) will increase the mean proliferative potential (we refer to it as longevity) and widen distribution of telomere lengths and thus individual cell longevities. While the wide distribution of telomere lengths is a common explanation of how both SITS and T-SCE extend longevity of proliferating cells, they will have opposing effects on the accumulation of mutations. Under the assumption of constant mutation rate, the number of mutations in individual cells will increase linearly with their longevity and thus the distributions in number of mutations will match the distribution of longevities. This means that while *SITS narrows the distribution of mutations* by letting the less damaged cells live longer and removing highly mutated cells, the *T-SCE widens the distribution of mutations* as it both widens the distribution of cell longevities and at the same time recombination makes cells “forget” about their history of exposure to DNA damage. Widening of the distribution is a highly undesirable effect: The wider is the distribution, the higher is the chance to arrive to potential “cancer progenitors” – cells with many more mutations than on average in a given population. Interestingly higher eukaryotes have acquired a rather involved machinery, e.g. shelterins [23], to put T-SCE under tight control.

**Figure 4:**
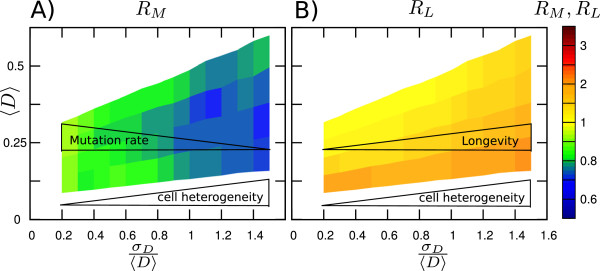
**Cell-to-cell variability is required for the beneficial effect of SITS.** The colorcoded are the ratios between the average SITS and TS mutation rates, *R*_*M*_, **(A)** and longevities, *R*_*L*_**(B)**. The ratios are shown as function of average level of DNA damage, <*D*> and cell-to-cell variability in DNA damage, *σ*_*D*_/<*D*>. The slice at *σ*_*D*_/<*D*>=1.5 would correspond to the ratios of the red and orange lines in Figure 3.

In Figure 4 we are investigating how the combined gain in mutation rate and longevity depends on the amount of cell-to-cell variability. To quantify the gain we have plotted the ratios of SITS to TS of mutation rates (*R*_*M*_) and longevities (*R*_*L*_), $R_{M}=\frac{<{\frac{\text{dM}}{\text{dt}}}_{\text{SITS}}>}{<{\frac{\text{dM}}{\text{dt}}}_{\text{TS}}>}$ and $R_{L}=\frac{<L_{\text{SITS}}>}{<L_{\text{TS}}>}$. (We choose normalized standard deviation, $\frac{\sigma_{D}}{<D>}$, also known as coefficient of variance, to quantify cell-to-cell variations.)

We find that the effect of SITS decreases as cells become more and more alike (decreasing $\frac{\sigma_{D}}{<D>}$). This suggests that stress induced telomere shortening and cell-to-cell variability in DNA damage inducing factors (e.g. metabolic load) are tightly interlinked.

Interestingly, the cells in *in vitro* cultures were shown to have widely variable doubling ability, even clonaly derived cells show distinct bi-modal distribution in number of doublings before senescence [24]. Passos et al. suggested that heterogeneity in telomere dependent senescence might stem from mitochondrial dysfunction [25].

## Conclusions

Telomeres are perfect oxidative stress sensors as they are particularly sensitive to oxidative DNA damage. The reason is two fold: First, they acquire DNA damage at a faster rate then the rest of genome. This is due to triple-G structure – present in telomeres of all eukaryotes – which are exquisitely sensitive to oxidative damage. Second, the repair of the damage is less efficient at telomeres [26] probably due to telomere binding proteins that restrict access to telomeric DNA (e.g. TRF2). These D͡NA-based sensors work in parallel with a complex apparatus of *protein-based* DNA damage sensors. In response to DNA damage kinases ATM, ATR and MRN complex are rapidly recruited and activated at the site of damage. If the damage is persistent the signal by these protein-based sensors will arrest cell cycle and activate pro-apoptotic p53 thus leading to non-telomeric senescence. Why did these two mechanism evolve to parallel each other? Is it important for telomere-induced senescence to relate to oxidative stress?

A possible explanation is that the two carry complementary functions: While p53-mediated response is dealing with persistent and acute damage, telomeres work as sentinels [27] and track the history of transient and repairable damage. As repair is imperfect it sometimes results in mutations. The ability of SITS to sense stress and track cell’s history of stress exposure allows it to “estimate” the amount of these mutations in individual cells. Our model shows that without sensing and estimating (as it would have been in a classical mechanism of TS due to end-replication problem) the mutation rate remains unaffected and mutations can only be limited as a direct consequence of limiting the life-span of the population. However, SITS – through its sensing ability – does alter the mutation rate (as shown in Figure 2), thus softening the coupling between longevity and the amount of accumulated mutations. Furthermore, not only it affects the mutation rate, it also increases longevity in a heterogeneous population of cells.

We find that for the SITS to have an effect cells must differ from each other. An interesting physiologically relevant example of heterogeneity is reported for the insulin producing pancreatic beta cells. The pancreatic beta cells have a highly variable glucose-sensing thresholds at which they start synthesizing insulin [28]. Cells with lower thresholds will, on average, synthesize more insulin, resulting in a higher metabolic load and thus higher ROS production. Our findings suggest that such specialization into “hard working, heavily damaged” and “lazy, undamaged” cells should delay population senescence and decrease mutations (consequently decreasing the chance for tumor to originate).

It will be interesting to apply our model to the particular case when DNA damage has a bi-modal distribution as it is probably the case in pancreatic beta-cells.

Another example where cells might experience highly variable DNA damage is NF-kB induction by tumor necrosis factor (TNF). The activity of Nf-kB has been directly linked to oxidative DNA damage through Nitrogen Oxygen Species (NOS) [29]. It appears that similarly to pancreatic beta cells, there is a high cell-to-cell variability in TNF sensing thresholds which results in high variability in NF-kB induction [30] (and consequently NOS induced DNA damage). Furthermore, it has recently been argued that cell-to-cell heterogeneity is practically unavoidable and can come from a multitude of sources – from stochastic heterogeneity in oxidative DNA damage due to e.g. variations in metabolic loads to deterministic heterogeneity due to variations in cell size, cell density, stage in cell-cycle, etc. [31].

At this stage we chose not to include the effects of stem cells and telomerase. While telomerase will extend the longevity within our model, we expect it to counteract SITS in reducing mutations at late timepoints. Expanding the model to include these points will allow one to address a number of exciting questions e.g. relating aging and cancer originating from stem cells. Another interesting aspect is that replicative senescence caused by critically short telomeres in some cell types is mediated by p53, such that damage in p53 restores replicative capacity [32]. While in our presented model the two processes– senescence caused by short telomeres and p53 mediated cell cycle arrest and apoptosis– act independently, it will be interesting to investigate how the interdependence of the two influence the response.

Our findings highlight the unique features of telomeric versus p53-mediated stress response and suggest that telomere mediated stress-sensing and cell-to-cell heterogeneity are crucial for reducing mutations and extending longevity.

## Competing interests

The authors declare that they have no competing interests.

## Authors’ information

Ala Trusina, Center for Models of Life, Niels Bohr Institute, University of Copenhagen, Blegdamsvej 17, Copenhagen 2100, Denmark. Email: trusina@nbi.dk

## Supplementary Material

Additional file 1 **Robustness of the results.** Supplementary Figure illustrating the robustness of the results. Values marked by circles represent values shown in the paper. **A)** and **B)** show that the results are robust to changes in system size. The converged (N=1600) average mutation rate, **A)**, and longevity, **B)**, show even stronger effect than for the system size (N=400) presented in the article. The results do not depend on the choice of the functional form of the mutation rate **C)** or the choice of the functional form for the apoptosis, **D)** and **E)**. The results are shown for the case with *σ*_*D*_/〈*D*〉 = 1.5 and 〈*D*〉=0.25. In all cases $R_{M}=\frac{<{\frac{\text{dM}}{\text{dt}}}_{\text{SITS}}>}{<{\frac{\text{dM}}{\text{dt}}}_{\text{TS}}>}<1$ and $R_{L}=\frac{<L_{\text{SITS}}>}{<L_{\text{TS}}>}>1$. Click here for file
