# Supplementary figures and images for: Stress induced telomere shortening: longer life with less mutations?
Source: BMC Syst Biol. 2014 Mar 1;8:27. doi: 10.1186/1752-0509-8-27 (PMC4015310; doi:10.1186/1752-0509-8-27)

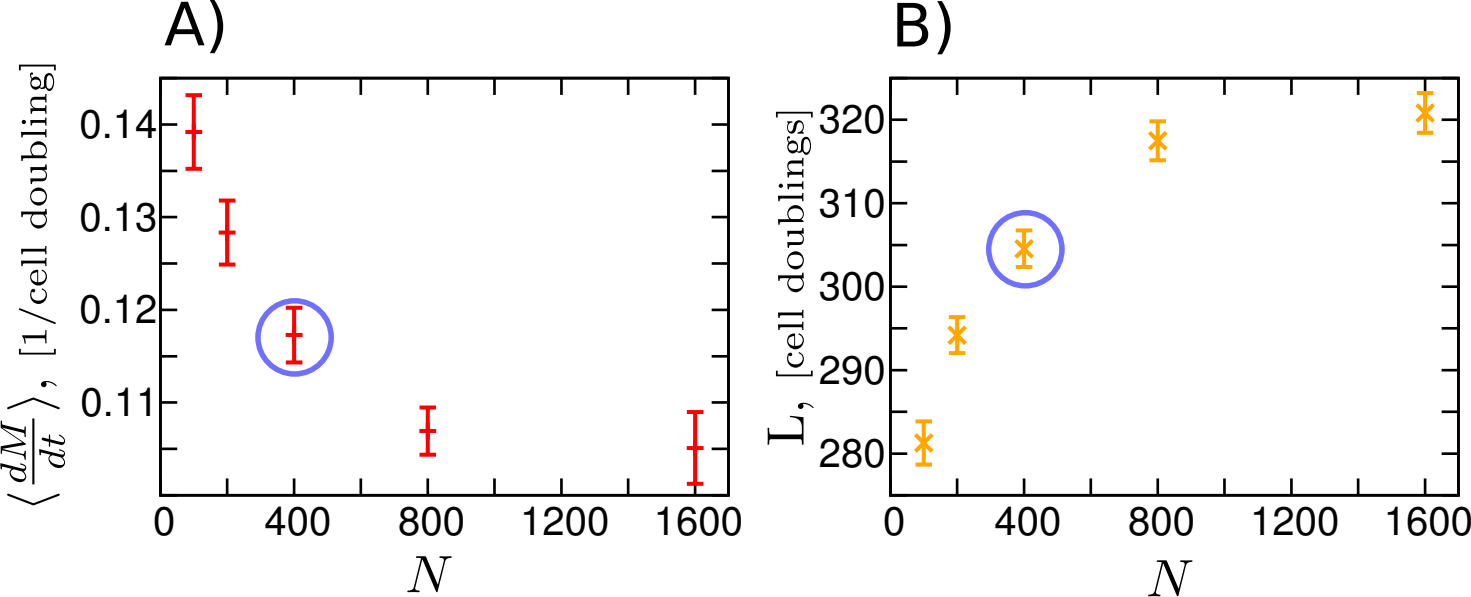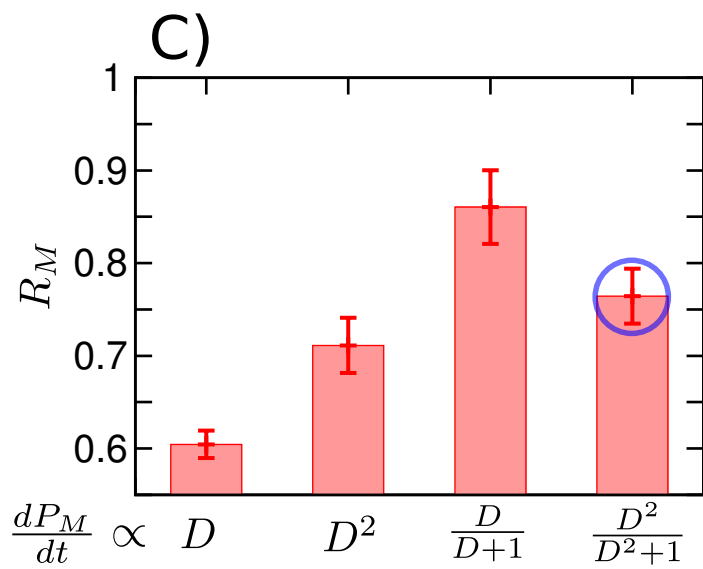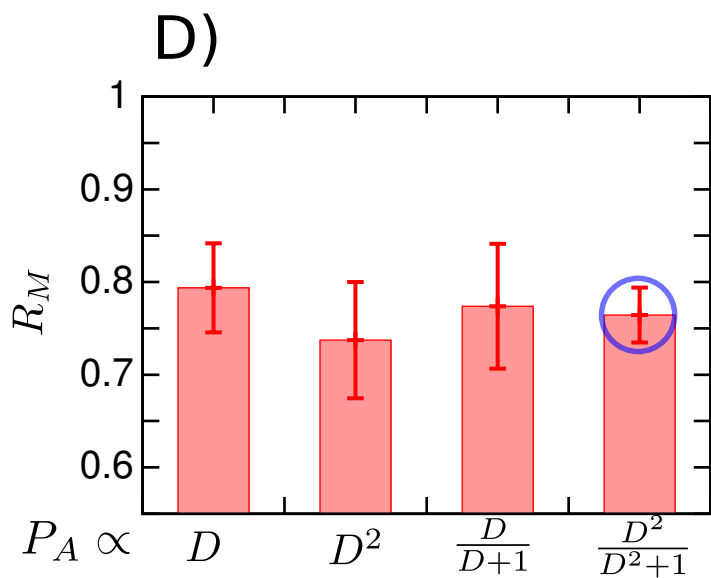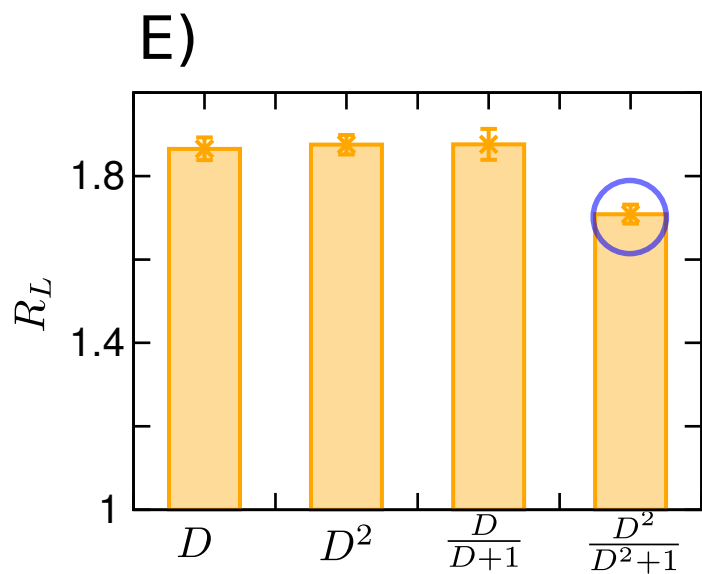

Supplement: Additional file 1 — Robustness of the results. Supplementary Figure illustrating the robustness of the results. Values marked by circles represent values shown in the paper. A) and B) show that the results are robust to changes in system size. The converged (N=1600) average mutation rate, A), and longevity, B), show even stronger effect than for the system size (N=400) presented in the article. The results do not depend on the choice of the functional form of the mutation rate C) or the choice of the functional form for the apoptosis, D) and E). The results are shown for the case with σD/〈D〉 = 1.5 and 〈D〉=0.25. In all cases RM=<1 and RL=>1. [file 1752-0509-8-27-S1.pdf]
